# Citral Sensing by TRANSient Receptor Potential Channels in Dorsal Root Ganglion Neurons

**DOI:** 10.1371/journal.pone.0002082

**Published:** 2008-05-07

**Authors:** Stephanie C. Stotz, Joris Vriens, Derek Martyn, Jon Clardy, David E. Clapham

**Affiliations:** 1 Howard Hughes Medical Institute, Department of Cardiology, Children's Hospital, Boston, Massachusetts, United States of America; 2 Department of Neurobiology, Harvard Medical School, Boston, Massachusetts, United States of America; 3 Department of Biological Chemistry and Molecular Pharmacology, Harvard Medical School, Boston, Massachusetts, United States of America; University of Southern California, United States of America

## Abstract

Transient receptor potential (TRP) ion channels mediate key aspects of taste, smell, pain, temperature sensation, and pheromone detection. To deepen our understanding of TRP channel physiology, we require more diverse pharmacological tools. Citral, a bioactive component of lemongrass, is commonly used as a taste enhancer, as an odorant in perfumes, and as an insect repellent. Here we report that citral activates TRP channels found in sensory neurons (TRPV1 and TRPV3, TRPM8, and TRPA1), and produces long-lasting inhibition of TRPV1–3 and TRPM8, while transiently blocking TRPV4 and TRPA1. Sustained citral inhibition is independent of internal calcium concentration, but is state-dependent, developing only after TRP channel opening. Citral's actions as a partial agonist are not due to cysteine modification of the channels nor are they a consequence of citral's stereoisoforms. The isolated aldehyde and alcohol *cis* and *trans* enantiomers (neral, nerol, geranial, and geraniol) each reproduce citral's actions. In juvenile rat dorsal root ganglion neurons, prolonged citral inhibition of native TRPV1 channels enabled the separation of TRPV2 and TRPV3 currents. We find that TRPV2 and TRPV3 channels are present in a high proportion of these neurons (94% respond to 2-aminoethyldiphenyl borate), consistent with our immunolabeling experiments and previous *in situ* hybridization studies. The TRPV1 activation requires residues in transmembrane segments two through four of the voltage-sensor domain, a region previously implicated in capsaicin activation of TRPV1 and analogous menthol activation of TRPM8. Citral's broad spectrum and prolonged sensory inhibition may prove more useful than capsaicin for allodynia, itch, or other types of pain involving superficial sensory nerves and skin.

## Introduction

Ion channels in the TRP family often act as sensors [1], detecting and responding to changes in pH, temperature, voltage, osmolarity, and exogenous molecules involved in taste, smell, and pheromone responses. The six TRP subfamilies (TRPC, TRPV, TRPM, TRPA, TRPP, and TRPML) encode putative six transmembrane secondary structures, with four subunits contributing to the tetrameric quaternary structure [2], [3]. Each subunit presumably contributes to a shared selectivity filter and ion-conducting pore similar to voltage-gated potassium channels [4]. TRP channels are present in almost all mammalian cell types, conducting primarily sodium and calcium from the extracellular milieu into the cell cytoplasm. The gating mechanisms of TRP channels are still poorly understood, but their activity is potentiated by the coincidence of multiple stimuli [5].

With a stronger and sweeter aroma than lemon, citral is a major component and the active ingredient of lemongrass oil, lemon peel, citronella, and palmarosa grass. Citral (3,7-dimethyl-2,6-octadienal) is composed of the double bond *trans* (geranial, citral A) and *cis* (neral, citral B) isomers. It is commonly used as a scent in perfumes and as a distinctive flavor in Southeast Asian cuisine. Approximately one-third of rat olfactory neurons respond to citral through the activation of unidentified endogenous receptors [6]. Widespread therapeutic effects have been attributed to citral at 1–100 µM in humans [7], [8]. Lemongrass is fed to cattle to reduce tick infestation, and at ∼250 µM, citral is lethal to insects [9], [10]. At millimolar concentrations, citral induces contact dermatitis in sensitized patients [11], [12]. The widespread effects of citral suggest multiple targets of action. TRP channels are excellent candidates for citral modulation since they are present in sensory cells and have known sensitivities to plant-derived compounds [13]–[16].

Here we have characterized the pharmacological effects of citral on several TRP channels known to be present in dorsal root ganglion neurons, including TRPV1–4, TRPM8, and TRPA1. Citral was found to activate and then inhibit TRP channel function. Irreversible citral inhibition was found to be state-dependent and calcium-independent. As citral is a mixture of two chiral isoforms, we investigated whether the isolated enantiomers of the aldehyde- and alcohol-containing compounds could explain these actions. To assess its potential usefulness in neurophysiology, we examined citral's action on native channels in freshly isolated dorsal root ganglion neurons. Capitalizing on its prolonged inhibition of TRPV1, we used citral as a tool to measure endogenous TRPV2 and TRPV3 currents. Finally, differences between rat and chicken TRPV1 sequences were exploited to identify a putative activation/inhibition-binding site for citral.

## Results

### Citral activates TRPV1, TRPV3, TRPM8, and TRPA1

**Figure 1:**
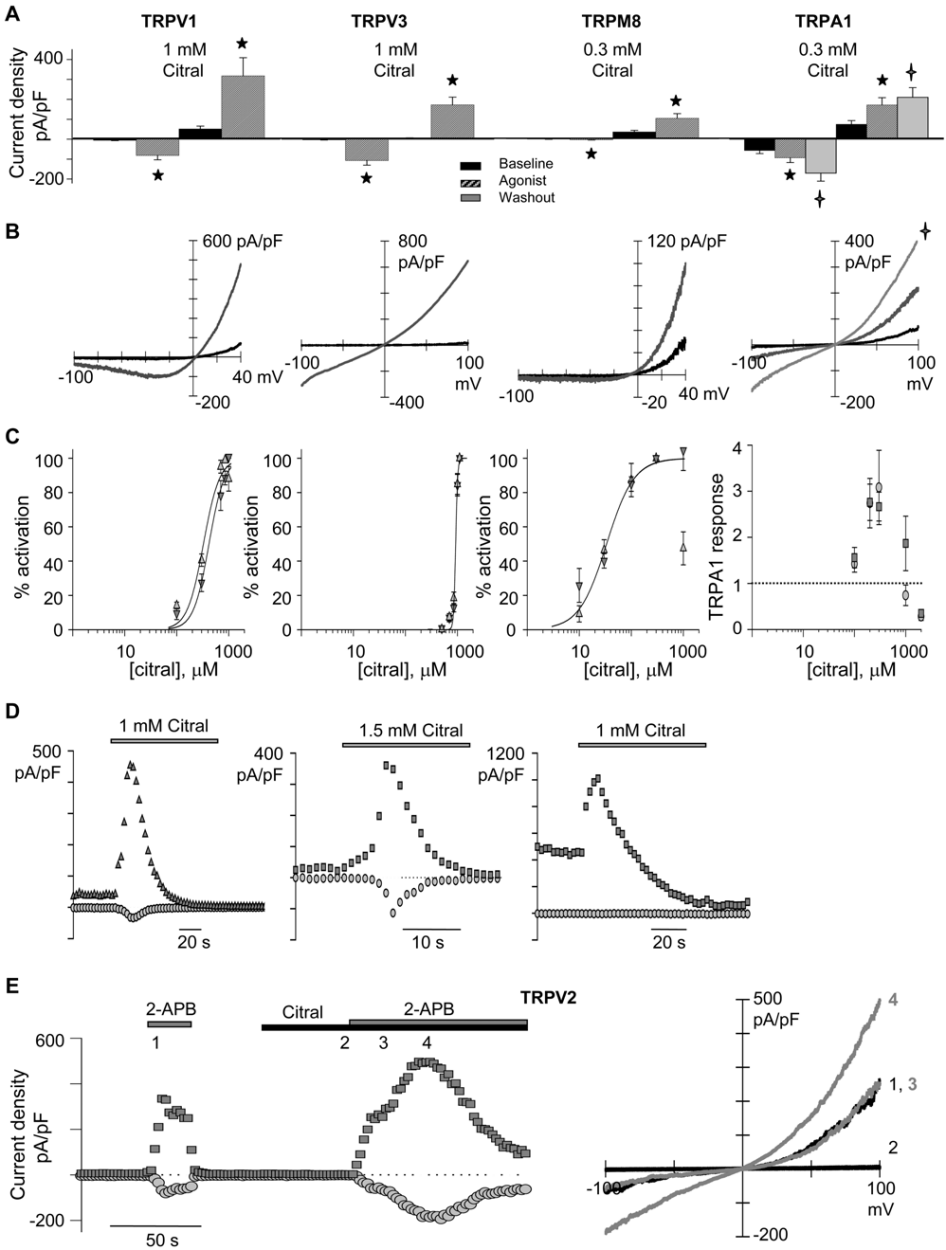
Citral is a partial agonist of rTRPV1, mTRPV3, rTRPM8, and rTRPA1. (A) Citral increased TRP channel activity above baseline currents (constitutive TRP+background+leak currents). TRPV1, TRPM8, and TRPA1 exhibit constitutive activity when highly expressed. TRPV1, TRPV3, TRPM8 are compared at −40 and +40 mV; TRPA1 at −100 and +100 mV). Citral increased inward (−40 mV) I_TRPV1_ by 14 fold, I_TRPV3_ by 38 fold, and I_TRPM8_ by 2 fold; I_TRPA1_ (−100 mV) increased by 3.1±0.8 fold. ★ indicates statistical differences between baseline and citral evoked current densities (unpaired Student's t-test, p<0.05 for TRPV1 (n = 8) and p<0.005 for TRPV3 (n = 15); paired Student's t-test, p<0.001 for TRPM8 (n = 11) and p<0.01 for TRPA1 (n = 9)). 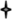 indicate statistical difference between baseline and citral washout current densities (unpaired Student's t-test, p<0.02 for TRPA1 (n = 9)). (B) Representative current-voltage relationships for citral-evoked TRPV1, TRPV3, TRPM8, and TRPA1 activity are presented. Baseline currents obtained prior to citral application are shown in black; citral-evoked TRP currents are in gray; 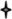 indicates TRPA1 currents measured following citral washout. (C) Dose response curves for citral activation of TRPV1, TRPV3, TRPM8, and TRPA1 are presented. All data are plotted with the same X-axis for comparison. Data were collected with nominal external calcium (∼10 µM) to reduce desensitization, with the exception of the TRPA1 recordings. Constitutive TRPA1 currents were normalized to 1. Data are presented at −100 mV (Ο), −40 mV (Δ), +40 mV (∇), and +100 mV (□). Averaged data were fit with the Hill equation (solid line; see Materials and methods, and Table 1 for apparent dissociation constants (K_D, app_) and Hill coefficients). For TRPV1 n = 8, TRPV3 n = 6–14, TRPM8 n = 4–11, and n = 4–9. (D) Citral inhibited the channels it activated. The continued presence of citral inhibited peak TRP currents by >50% within 6 s for TRPV3 (n = 6), and 14 s for TRPV1 and TRPM8 (n = 9 and 7, respectively). Note that citral also inhibited constitutive TRP current. Data are presented at −100 mV (Ο), +40 mV (∇), and +100 mV (□). (E) Sustained citral inhibition of TRPV2 required that the channels be open. A representative time course (−100 mV (Ο) and +100 mV (□)) and corresponding I-V relations for TRPV2 activation by 2-aminoethyldiphenyl borate and inhibition by citral is presented. Note that TRPV2 current did not activate with citral application (n = 4). Citral inhibition of TRPV2 occurred once the channels entered their open (conducting) state, evoked by the addition of 2-aminoethyldiphenyl borate. Note that citral also potentiated the 2-aminoethyldiphenyl borate response before inhibition developed.

Citral was applied to heterologously expressed TRP channels known to be present in sensory neurons while assessing their activity via whole-cell patch clamp. Citral significantly increased TRPV1, TRPV3, TRPM8, and TRPA1 (Fig. 1A and B) currents, but not TRPV2 (Fig. 1E), TRPV4, or background currents in nontransfected cells (data not shown). Citral was less potent (Fig. 1C) and less efficacious than most well known TRP channel agonists (Table 1); citral's order of potency for activation was TRPM8>TRPV1>TRPA1>TRPV3. At high agonist concentrations, inhibition often began to develop before activation reached steady state, obscuring the peak current evoked for TRPV1, TRPV3, and TRPM8 (Fig. 1D). The apparent dissociation constants (K_D, app_) for citral activation were essentially voltage-independent (Fig. 1C). Evoked TRPV3 current exhibited an extraordinarily steep dependence on citral concentration (Hill coefficient of 22.3, compared to 1.6 for TRPM8 and 2.7 for TRPV1). The process underlying TRPV3's unusual sensitization may account for this high apparent cooperativity [17].

**Table 1:** Affinity of sensory TRP channels for citral.

TRP channel	Activation K_D, app_	Hill coefficient	Agonist efficacy	Inhibition K_D, app_	Hill coefficient
TRPV1	465 µM (−40 mV)	2.7	I_pH5_/I_citral (1 mM)_ 28±8 fold larger (n = 3; −40 mV)	187 µM	1.3
	417 µM (+40 mV)	2.9			
TRPV2	Ø		Ø	534 µM	2.4
TRPV3	926 µM	22.3	I_2-APB (100 µM)_/I_citral (1 mM)_ 25±14 fold larger (n = 6; −40 mV)	465 µM	4.0
TRPV4	Ø		Ø	32 µM	0.9
TRPM8	33.5 µM	1.6	I_menthol (30 µM)_/I_citral (300 µM)_ 74±28 fold larger (n = 5; −40 mV)	241 µM (−40 mV)	2.0
				188 µM (+40 mV)	1.7
TRPA1	threshold ∼200 µM		I_AITC (300 µM)_/I_citral (300 µM)_ 1.5 fold larger (n = 27 and 9; −100 mV)	254 µM	1.0

Ø - not activated

### Agonist-evoked TRP channel activity is increased and potentiated by citral

**Figure 2:**
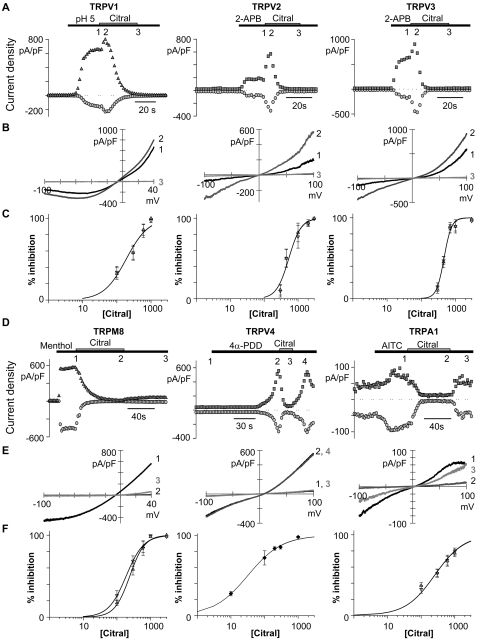
Citral inhibits agonist evoked TRP currents. Data were collected with nominal external calcium to reduce desensitization (except for TRPA1). Note the absence of TRPV1–3, and TRPM8 desensitization in the continued presence of low pH solution, 2-aminoethyldiphenyl borate, or menthol. Citral (1 mM) increased TRPV1- (1.8±0.3 fold, −100 mV; 1.6±0.2 fold at +40 mV; n = 8), TRPV2- (2.5±0.4 fold, −100 mV; 2.2±0.2 fold, +100 mV; n = 10) and TRPV3- (1.7±0.3 at −100 mV; 1.3±0.1 fold at +100 mV; n = 7) agonist-evoked currents prior to onset of its inhibitory action. Agonist-induced TRPM8-, TRPV4-, and TRPA1- currents were not increased by the addition of citral (n = 4, 9, and 10). Symbols indicate data analyzed at −100 mV (○), −40 mV (∇), +40 mV (Δ) or +100 mV (□). (A and D) Representative time courses are presented for agonist-specific activation and citral inhibition of TRPV1–4, TRPM8, and TRPA1. Citral inhibition was prolonged for TRPV1–3 and TRPM8; 30 seconds after citral washout pH5 evoked only a 0.2±0.1% increase in TRPV1 current (n = 5). After 60 seconds washout, a 4±2% increase in TRPM8 current was evoked by menthol (n = 3). In contrast, inhibition of TRPV4 and TRPA1 reversed with citral washout; at 30s, 60±16% of TRPA1 current activation by allyl isothiocyanate was recovered (n = 5). (B and E) TRP currents recorded at time points indicated in A and D (numbers) as a function of voltage. (C and F) Dose response curves for citral block of agonist-evoked TRPV currents. Agonist concentrations for TRPV1–4, TRPM8 and TRPA1 were pH5, 700 µM 2-aminoethyldiphenyl borate, 10 µM 4α-phorbol-12, 13-didecanoate, 1 mM menthol, and 300 µM allyl isothiocyanate. Averaged data were fit with the Hill equation (solid lines, see Materials and methods, and Table 1 for K_D, app_ and Hill coefficients). For TRPV1 n = 5–8, TRPV2 n = 5–8, TRPV3 n = 4–9, TRPV4 n = 6–10, TRPM8 n = 3–7, and TRPA1 n = 6–9.

We next examined whether citral evoked an additive response in TRP channels activated by other established agonists (Fig. 2). Citral initially increased the agonist-evoked responses of TRPV1, TRPV2, and TRPV3 (Fig. 2A, B). In contrast, agonist activation of TRPM8, TRPV4, and TRPA1 was not increased or potentiated by citral (Fig. 2D, E). This lack of enhancement may reflect an absence of a citral activation binding site, or saturation of a common binding site by menthol (30 µM), 4α-phorbol-12, 13-didecanoate (10 µM), and allyl isothiocyanate (300 µM), respectively.

### Citral inhibition of TRPV1–4, TRPM8, and TRPA1

Citral inhibited the agonist-evoked activity of TRPV1–4, TRPM8, and TRPA1 (Fig. 2C, F); citral's order of potency for inhibition was TRPV4>TRPV1>TRPM8∼ TRPA1∼TRPV3∼ TRPV2 (Table 1). Inhibition was essentially voltage-independent in all cases.

The process of citral inhibition was distinguished from previously described TRP channel calcium-dependent desensitization [18] using experimental conditions that limited the external calcium concentration to ∼10 µM and tightly buffered the internal calcium concentration to <100 nM (10 mM BAPTA (1,2-bis(o-aminophenoxy)ethane-N,N,N',N'-tetraacetic acid)). These conditions prevented calcium-dependent desensitization, stabilizing the responses of TRPV1 to pH 5 solution, TRPV2 and TRPV3 to 2-aminoethyldiphenyl borate, and TRPM8 to menthol (Fig. 2A, D). Citral inhibition developed despite highly buffered internal calcium, suggesting an underlying calcium-independent mechanism.

For TRPA1, the overlapping apparent dissociation constant of citral activation and inhibition resulted in a bell-shaped dose-response curve (Fig. 1C). Since citral washout significantly increased TRPA1 activity (Fig. 1A, B; Fig. S1A), we surmised that citral evoked sustained channel activity that was then transiently blocked at higher citral concentrations. Reapplication of citral reduced TRPA1 current (tachyphylactic response; Fig. S1B).

### Recovery from citral inhibition

Citral inhibition of agonist-evoked TRPV1, TRPV2, TRPV3, and TRPM8 currents was essentially irreversible (Fig. 2A, D). In contrast, 4α-phorbol-12, 13-didecanoate -evoked TRPV4 and allyl isothiocyanate-evoked TRPA1 currents rapidly reversed.

The expected recovery rate of channel activity from diffusible (soluble) blockers can be estimated using the diffusion rate (10^8^ M^−1^s^−1^) as the upper limit for the rate of inhibition and the apparent dissociation constant values obtained from the dose response curves [19]. For compounds with µM affinity, complete recovery from open channel block should be evident within seconds after washout. The recovery rates of TRPV1–3 and TRPM8 were at least ten times longer than predicted, suggesting that citral inhibition was not due to block of open channels via free diffusion in saline solution. The recovery times of TRPV4 and TRPA1 suggest, however, that these channels are inhibited by citral through reversible binding and diffusion.

### Irreversible citral inhibition develops once TRPV2 channels enter their open state

The state dependence of irreversible citral inhibition was determined for TRPV2, a channel with negligible constitutive currents and resistant to citral activation. Pretreatment with citral did not prevent TRPV2 activation by 2-aminoethyldiphenyl borate (Fig. 1E), but inhibited the channels once they entered their active (conducting) state. Similarily, TRPV1, TRPV3 and TRPM8 likely enter the open state before irreversible citral inhibition develops. High citral concentrations (∼1 mM) initially activated then inhibited these channels (Fig. 1D). Once inhibition developed, increasing citral concentration did not elicit currents. However, agonists of greater efficacy (pH 5, 2-aminoethyldiphenyl borate, or menthol) were still able to evoke currents (data not shown). Thus, citral inhibited only the channels it had initially activated. This finding suggests that the binding sites underlying the respective activation/inhibition mechanisms are not identical.

### Effect of isolated citral enantiomers and related alcohols on TRPs

**Figure 3:**
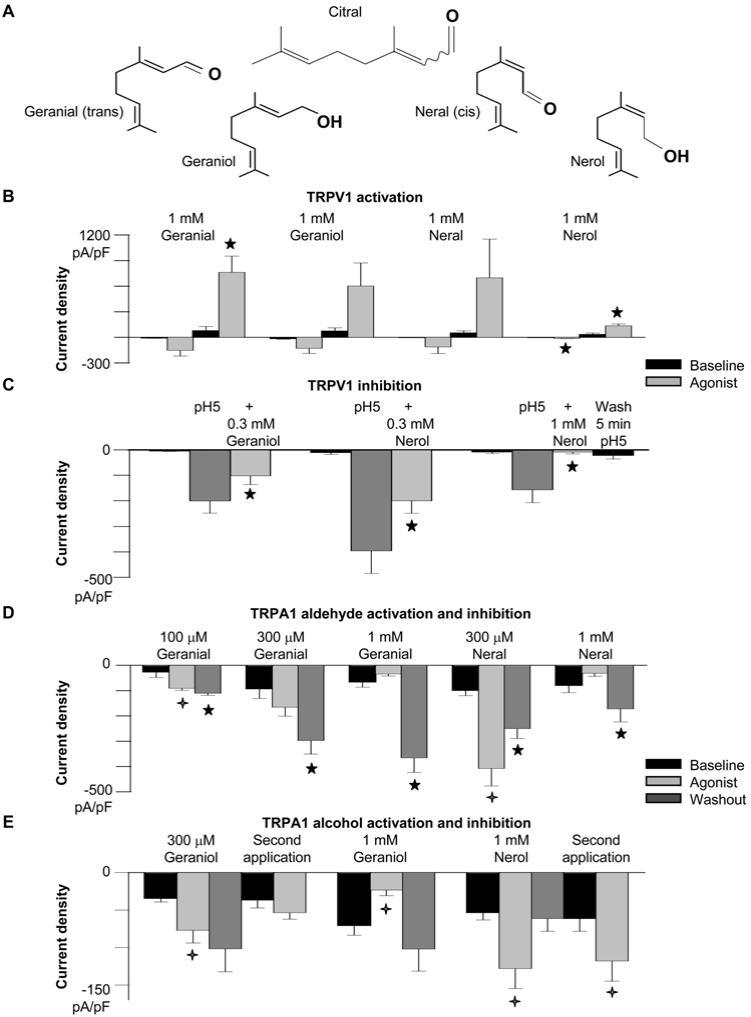
Citral *cis* and *trans* enantiomers and alcohol derivatives evoke and inhibit TRPV1 and TRPA1 currents. (A) The chemical structures of citral, geranial, geraniol, neral, and nerol are illustrated. (B) The TRPV1 current was weakly activated by the citral aldehyde (geranial, neral) and alcohol (geraniol, nerol) isomers (analyzed at −40 mV and +40 mV). Significant differences are indicated by ★ (paired Student's t-test, p<0.05 for geranial (n = 4), and nerol at +40 mV; paired Student's t-test, p<0.005 for nerol at −40 mV (n = 3); geraniol (n = 3); neral (n = 5)). (C) Geraniol and nerol inhibit pH 5-evoked TRPV1 current (analyzed at −40 mV). Significant differences are indicated by ★ (paired Student's t-test, p<0.005 for 300 µM geraniol (n = 6) and p<0.05 for 300 µM nerol (n = 5); unpaired Student's t-test, p<0.05 for 1 mM nerol (n = 4)). 1 mM nerol completely and irreversibly inhibited the pH 5-evoked TRPV1; <1% of TRPV1 current was recovered 5 minutes after washout in 3 of 4 cells). (D) Low concentrations (0.1–0.3 mM) of the aldehyde enantiomers activated TRPA1 currents, while only inhibition was evident with higher concentrations (1 mM) until washout. Subsequent applications inhibited TRPA1 current. The external calcium concentration was 2 mM. Data are compared at −100 mV (−120 mV for 300 µM geranial and neral). Significant differences between baseline and the aldehyde responses are indicated by 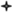 (unpaired Student's t-test, p<0.05 for 100 µM geranial (n = 3); p<0.005 for 300 µM neral (n = 5)). Significant differences between baseline and aldehyde washout are indicated by ★ (unpaired Student's t-test, p<0.05 for 100 µM and 300 µM geranial (n = 3 and 4, respectively), p<0.01 for 1 mM geranial (n = 3) and 300 µM neral (n = 5); paired Student's t-test, p<0.05 for 1 mM neral (n = 4)). (E) Summary of the TRPA1 response to citral-related alcohols (−100 mV). 300 µM geraniol and 1 mM nerol increased TRPA1 current (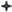, unpaired Student's t-test, p<0.05 for both, n = 5–9), while 1 mM geraniol blocked 70% of the inward TRPA1 current (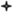, unpaired Student's t-test, p<0.01, n = 9). A second application of 1 mM nerol also increased TRPA1 current (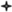, 2 fold increase, paired Student's t-test, p<0.05, n = 4).

Citral contains the chiral enantiomers, neral (*cis*) and geranial (*trans*; Fig. 3A). Were citral's partial agonist effects due to the opposing actions of the aldehyde enantiomers? To generate the aldehydes, we oxidized the commercially available alcohols, nerol, and geraniol (see Materials and methods). Both aldehydes and their related alcohols (neral, nerol, geranial, and geraniol) weakly activated TRPV1 (Fig. 3B), TRPV3, TRPM8 (Fig. S2), and TRPA1 (Fig. 3D, E). As with citral, higher concentrations of the compounds also inhibited the currents they induced (Fig. 3D, E; Figs. S3 and S4,). Thus, citral's inverse actions are not explained by opposing activation/inhibition by enantiomers.

The fact that some of the compounds are alcohols also constrains the potential mechanisms underlying sustained inhibition of TRPV1–3 and TRPM8. Inhibition of TRPV1 by 1 mM nerol (100%) was sustained after 5 minutes washout (Fig. 3C). Since the side chain group of nerol is not reactive, it is unlikely that inhibition is mediated by covalent modification of TRPV1. Geraniol and nerol inhibited TRPV1 currents evoked by pH 5 solution less effectively than citral, however; currents decreased 45% and 50% in the presence of 300 µM geraniol and nerol respectively (Fig. 3C). We suspect that the slightly higher potency of the aldehyde is related to its higher lipophilicity. The effectiveness of pharmaceutical agents targeting membrane proteins correlates well with partition coefficients [20], presumably due to their ability to interact with hydrophobic regions of the protein and/or plasma membrane.

The alcohol compounds reversibly and repetitively increased constitutively active TRPA1 currents (Fig. 3E), while citral (Fig. S1), neral, and geranial (data not shown) increased TRPA1 activity only upon first application. Similarly, menthol was found to repetitively activate TRPA1 currents [21]. Covalent modification of TRPA1 amino terminal cysteines has been recently proposed as the molecular basis of its activation by diverse electrophiles [22], [23]. During activation, the aldehydes likely bind covalently to channel cysteine residues; thus repetitive activation is not observed. Geraniol and nerol, however, are not electrophilic, and it is improbable that they modulate TRPA1 activity through covalent modification. We suspect that structural similarities and hydrophobicity are more liable to underlie a common activation mechanism for these aldehyde and alcohol enantiomers.

### Further studies addressing possible mechanisms underlying sustained TRP channel inhibition

Could sustained citral inhibition reflect increased endocytosis or a reduction in the rate of channel incorporation? Confocal and TIRF imaging of enhanced green fluorescent protein-tagged TRPV3 revealed no significant changes in the surface localization of 2-aminoethyldiphenyl borate -activated channels (data not shown). Furthermore, surface biotinylation experiments indicated that the relative number of membrane expressed 2-aminoethyldiphenyl borate -activated TRPV2 channels were not altered by citral treatment (data not shown). Lastly, citral treatment did not cause macroscopic disruptions in subunit interactions (Fig. S5A); hemagglutinin A- and enhanced green fluorescent protein-tagged TRPV2 subunits co-immunoprecipitated both in presence and absence of citral. We predict that sustained citral inhibition reflects a series of complex interactions between the compound, membrane, and channel that will best be resolved by the attainment of high-resolution structure and accompanying mutagenesis studies.

### Citral can be used to identify the TRPV1–3 contributions to native currents

**Figure 4:**
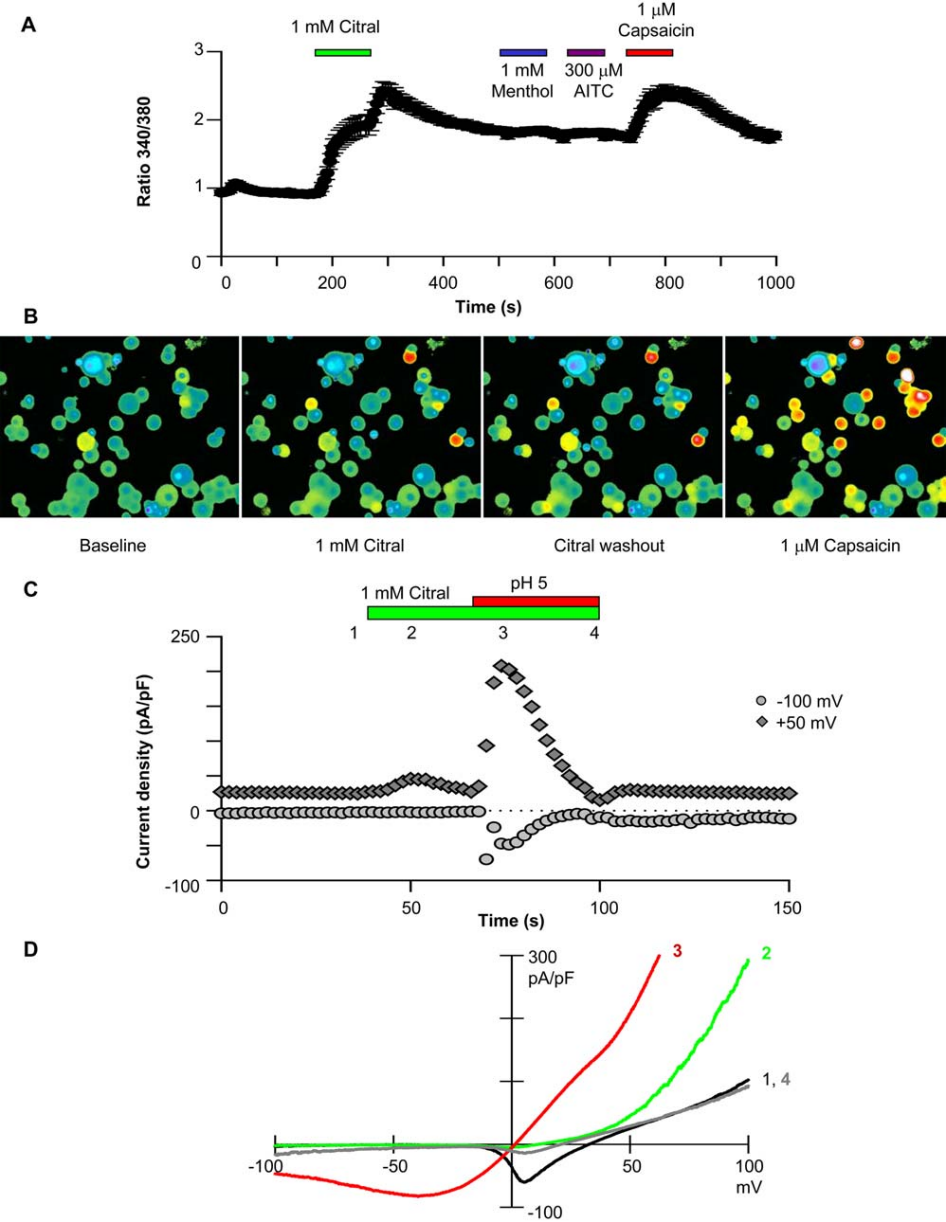
Citral evokes currents consistent with TRPV1 activity and increases the internal calcium concentration in capsaicin/pH 5-responsive dorsal root ganglion neurons. (A) The average time course of calcium entry into dorsal root ganglion neurons responsive to both citral and capsaicin (12 of 35 capsaicin responders) is shown. Changes in internal calcium concentration were plotted as the ratio of fura-2 intensity measured at 340 and 380 nm (see Materials and methods). (B) Images of the dorsal root ganglion neurons before agonist application, during 1 mM citral perfusion, during citral washout, and after 1 µM capsaicin application are presented. (C) A typical time course of dorsal root ganglion currents activated by 1 mM citral and pH 5 solution is presented. Citral transiently activated outward dorsal root ganglion currents and suppressed voltage-gated ion channels. Citral further inhibited pH 5-evoked dorsal root ganglion currents. A small increase in inward dorsal root ganglion current was noted after pH5/citral washout. (D) Dorsal root ganglion neuron current-voltage relations obtained from voltage ramps at the time points indicated in C.

**Figure 5:**
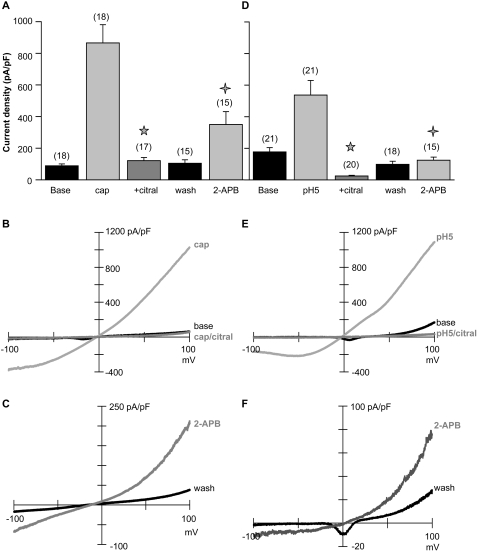
Citral irreversibly inhibits dorsal root ganglion TRPV1-like currents activated by 1 µM capsaicin (A, B) or pH 5 solution (D, E). Statistical differences between agonist±citral current densities are indicated by ★ (unpaired Student's t-test, p<0.000005 in A and D). Potentiation of dorsal root ganglion TRPV1-like current prior to inhibition was greater with pH 5 activation (1.5±0.1 fold; p<0.0005, paired Student's t-test; n = 20) than with capsaicin activation (1.1±0.03 fold; n = 17; data not shown). After inhibition of TRPV1 currents, 2-aminoethyl diphenyl borate subsequently activated currents consistent with TRPV2 or TRPV3 activity (A, D). Statistical differences between wash and 2-aminoethyldiphenyl borate evoked current densities are indicated by 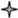 (unpaired Student's t-test, p<0.01 in A; paired Student's t-test, p<0.0001 in D). Holding potential = 0 mV. (B, C; E, F) Representative current-voltage relationships illustrating citral inhibition and 2-aminoethyldiphenyl borate activation from a neuron initially stimulated with capsaicin (B, C) or pH5 (E, F).

Citral increased the internal calcium concentration in 12 of 35 freshly isolated dorsal root ganglion neurons responsive to capsaicin (Fig. 4, B). Citral washout further increased the internal calcium concentration in these dorsal root ganglion cells. In two cells unresponsive to citral application, washout also elevated the internal calcium concentration, consistent with the presence of TRPA1 channels (see Fig. 1A, B and Fig. S1; TRPA1 is expressed in ∼30% of TRPV1-positive dorsal root ganglion neurons [24]). In patch clamp recordings of isolated dorsal root ganglion neurons, slow voltage-clamp ramps from a holding potential of 0 mV minimized current contributions from fast, strongly voltage- and time-dependent channels ([25]; note that the fast inward current, primarily voltage-gated sodium channels, was partially blocked by 1 mM citral; Fig. 4D). Focusing on TRP currents, citral increased outward currents by 2.7±0.5 fold, while pH 5 solution increased outward currents by 6.3±2.2 fold (n = 8; Fig. 4C, D). The citral induced calcium influx and outward currents are likely attributable to TRPV1 or TRPV3 activity, since TRPM8 is present in capsaicin/pH unresponsive dorsal root ganglion cells [24], [26]. Citral rapidly inhibited dorsal root ganglion TRPV1-like currents evoked by capsaicin (94% block; Fig. 5A, B) or pH 5 solution (100% block; Fig. 5D, E). Inhibition was practically irreversible; only 3±1.5% (n = 8) of the capsaicin response recovered 5 minutes after washout. Citral at pH 5 also blocked a component of the constitutive current (79% of initial outward current was inhibited; Fig. 5D, E). A TRPA1-like current was not apparent after citral washout (Fig. 5A, C, D, F).

Based on *in situ* hybridization data [17], our laboratory reported that TRPV3 was ubiquitously expressed in dorsal root ganglion neurons. Camphor primarily activated a TRPV1-like current, however, suggesting that TRPV3 channels were present in lower density per cell [25]. In the present experiments, 2-aminoethyldiphenyl borate evoked currents in 27 of 30 cells in which TRPV1 was inhibited (3±0.4 fold increase; unpaired Student's t-test, p<0.005; Fig. 5A, C, D, F). We hypothesized that the 2-aminoethyldiphenyl borate current was due primarily to TRPV2 and TRPV3 activity and sought to determine their relative contributions. We used the criteria that TRPV2 is responsible for currents not potentiated by repetitive 2-aminoethyldiphenyl borate, while sensitization and camphor activation typify TRPV3 currents [15], [25], [27].

**Figure 6:**
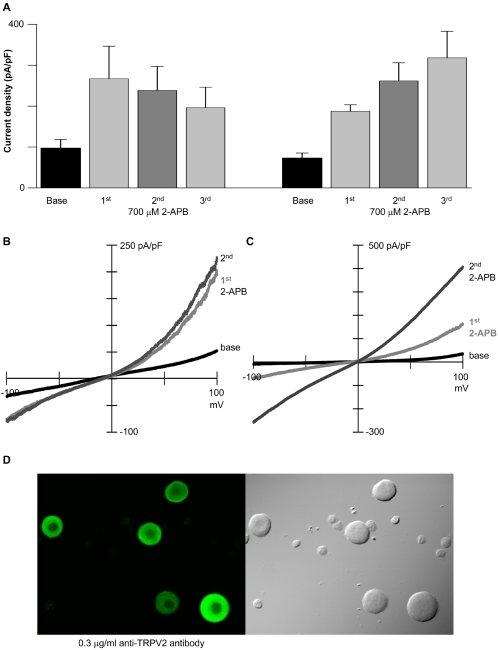
Citral inhibition of I_TRPV1_ reveals the expression of TRPV2 and TRPV3 in acutely isolated dorsal root ganglion neurons. (A) Two patterns of response to repetitive 2-aminoethyldiphenyl borate application were noted within the dorsal root ganglion population following citral block of capsaicin-activated currents; dorsal root ganglion currents decreased in six neurons (left), and increased in the remaining neurons (right; n = 6; +100 mV). Holding potential = 0 mV. (B) Representative dorsal root ganglion currents from a neuron not potentiated by repeated 2-aminoethyldiphenyl borate application are shown. (C) Representative dorsal root ganglion currents from a neuron potentiated (2.3 fold; +100 mV) by repeated 2-aminoethyldiphenyl borate application are shown. (D) TRPV2 immunoreactivity in dorsal root ganglion neurons stained with 0.3 µg/ml anti-TRPV2 antibody+Alexa 488 secondary is shown (left; see *Figure S5* for controls). Differential interference contrast image (right).

In the first 2-aminoethyldiphenyl borate responsive population (six of twelve neurons), current amplitude decreased slightly with repetitive 2-aminoethyldiphenyl borate application (Fig. 6A, B). Camphor did not activate a significant conductance, but blocked background currents in three of these dorsal root ganglion neurons (data not shown). Thus, in this population 2-aminoethyldiphenyl borate primarily activated currents consistent with TRPV2 activity. In the second population, neurons were potentiated 1.7±0.3 fold by repetitive 2-aminoethyldiphenyl borate application (Fig. 6A, C), and camphor evoked a small increase in outward current (1.3 fold±0.07; paired Student's t-test, p<0.01; data not shown), consistent with TRPV3 activity. Thus, TRPV2 or TRPV3 activity dominated the 2-aminoethyldiphenyl borate response in citral-inhibited TRPV1-positive neurons. However, TRPV2 activity in a predominantly TRPV3-expressing cell, or low TRPV3 activity in a predominantly TRPV2-expressing cell, cannot be ruled out by these methods.

To confirm the expression of TRPV2 in juvenile rat dorsal root ganglion neurons, we stained acutely isolated cells with an anti-TRPV2 antibody generated to the carboxyl-terminus of the protein. Significant TRPV2 expression was observed in these neurons (63 of 67 cells; Fig. 6D; see Fig. S5C and D for controls). The TRPV2 immunoreactivity was much stronger in dorsal root ganglion neurons isolated from juvenile (postnatal day 10–20) rats than previously reported for adult rats [28]–[30]. Our data confirm widespread expression of TRPV2 in dorsal root ganglion neurons, and the pharmacological data suggest that TRPV2 is present in cells that also express TRPV1.

### Citral and capsaicin share a binding region for TRPV1 activation

**Figure 7:**
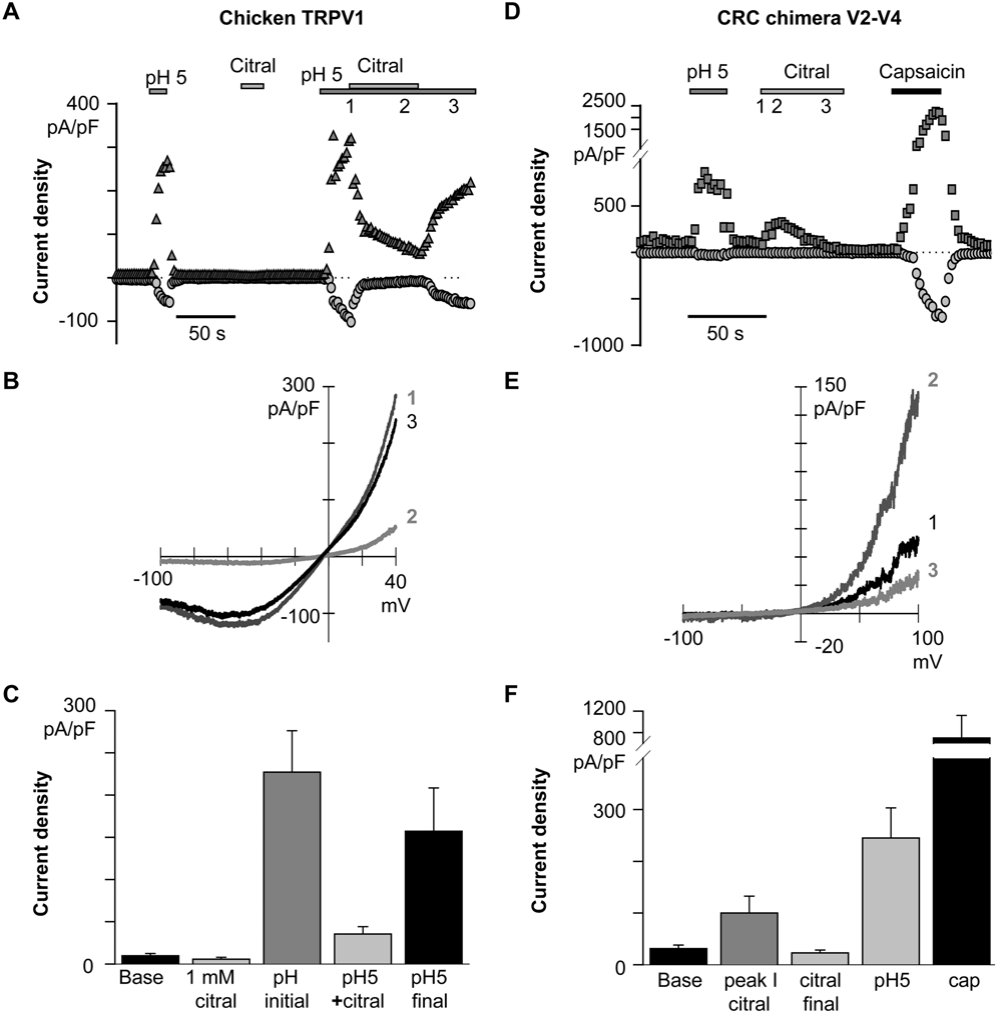
The second through fourth transmembrane region of TRPV1 is implicated in citral activation. Symbols indicate data analyzed at −100 mV (○), +40 mV (Δ) or +100 mV (□). (A) Chicken TRPV1 current is not activated by citral. However, agonist-evoked activity was reversibly blocked. A representative time course is presented. (B) Chicken TRPV1 (cTRPV1) current-voltage relations, obtained at the time points indicated in A, are presented. (C) Summary of the cTRPV1 data (+40 mV; n = 6). (D) Citral activation was restored in the CRC chimera (a construct introducing the rat (r) capsaicin-binding region (transmembrane segments two through four) into the chicken (c) TRPV1 scaffold). Thus, the mammalian capsaicin-binding region was required for citral sensitivity. Note that the citral-evoked CRC current was transient. A representative time course is presented. (E) CRC chimera current-voltage relations are presented corresponding to the time points indicated in D. (F) Summary of CRC chimera current densities analyzed at +100 mV (n = 6).

Transmembrane segments two through four have previously been implicated in the binding-dependent activation of TRPV1 by capsaicin, and TRPM8 by menthol and icilin [31]–[33]. Jordt and Julius (2002) further demonstrated that the avian TRPV1 is capsaicin-insensitive due to transmembrane segment two through four sequence differences. Similarly, we found citral did not activate the capsaicin-insensitive chicken homolog of TRPV1 (cTRPV1), but reversibly inhibited currents activated by pH 5 solution (Fig. 7A–C). Substituting the cTRPV1 transmembrane segment two through four region with the corresponding rat sequence (CRC V2–V4 chimera [31]) restored citral activation. Citral transiently evoked TRPV1 CRC V2–V4 currents (Fig. 7D–F), suggesting that the domain necessary for activation/binding had been restored. Interestingly, the capsaicin-insensitive rat TRPV1 tyrosine to alanine point mutant at residue 511 [31] remained responsive to citral (data not shown). Thus, citral's interaction with TRPV1 is not identical to that of capsaicin, although the transmembrane segment two through four region of the voltage-sensor/ligand domain appears to be a common modulatory region for several TRP channels.

## Discussion

From bacteria to man, food detection and acquisition is fundamental to survival. All the senses are tasked to make decisions about ingestion, and TRP channels, abundantly located in the nerve endings of the mouth, tongue, and nose, play an important role in chemical sensing. Living in symbiosis with animals, plants evolved chemicals that specifically attract animals for seed dissemination or dissuade them from harmful activity. Citral is a plant compound with widespread biological effects. Like other plant compounds such as capsaicin, menthol, allicin, carvacrol, eugenol, and vanillin [1], [14], [16], [26], [34]–[36], citral activates and modulates TRP ion channels. We have shown that citral acts as a partial agonist of TRPV1, TRPV3, TRPM8, and TRPA1. Probably the most pharmacologically relevant effect described here is citral's prolonged inhibition of TRPV1–3 and TRPM8 following activation. Citral (in lemongrass) is often combined with capsaicin in culinary preparations such as Thai food. Citral should squelch the ‘hot’ capsaicin-induced sensation; initially potentiating, then suppressing it. Citral's primary potency on TRPM8 may explain its initial ‘cool’ sensation, and citral may modulate a plethora of skin, nerve, and epithelial cell-mediated sensory responses via the TRPV2, TRPV3, TRPV4, and TRPA1 channels.

Citral interacts with regions circumscribed by the second through fourth transmembrane helices of TRPV1. This region contains residues essential for capsaicin activation of TRPV1 [31] and menthol activation of TRPM8 [33]. Citral activated the capsaicin-insensitive tyrosine to alanine TRPV1 point mutant at residue 511, suggesting that the binding sites for capsaicin and citral are not identical. Camphor, which shares the same molecular formula (C_10_H_16_O) as citral, did not require the region to activate TRPV1 [25]. The structures of citral and camphor, however, are quite distinct; citral is an aldehyde with an unsaturated hydrocarbon chain, while camphor is a bicyclic ketone. More studies will be needed to determine whether the second through fourth transmembrane region is a general ligand or toxin/TRP channel interaction domain.

Chirality is often an important feature of drug efficacy; the dihydropyridine-sensitive (L-type) voltage-gated calcium channel blocker Bay-K 8644 (-)-S enantiomer activates, while the (+)-R enantiomer blocks these channels [37]. Citral is both an activator and inhibitor of TRPV1, TRPV3, TRPM8, and TRPA1, but these opposing actions cannot be attributed to the *cis* and *trans* enantiomers. Neral and geranial also activated and inhibited the TRP channels, as did their alcohol derivatives. Sustained inhibition of TRPV1–3, and TRPM8 developed subsequent to channel activation. As we have shown, this inhibition is not consistent with simple open channel block, calcium -dependent desensitization, cysteine modification, or suppression of channel surface expression.

Exogenous small molecules modulate target proteins via electrostatic, dipole, hydrogen donor/acceptor, aromatic stacking (π – π), or lipophilic types of interactions. Based on the similarity of citral aldehyde and alcohol enantiomers' actions, citral's log P (oil/water) coefficient of ∼three, the generally slow time course of inhibition, and limited data from the chimeras, we hypothesize that citral's primary mechanisms involve hydrophobic interactions with the channel protein and/or phospholipid-protein interface. As for all other exogenous TRP channel modulators, the mechanisms for citral's complex partial agonist effects are not currently understood. Deeper understanding of small molecule effects on TRP channel gating will require high resolution structural information, structurally informed mutagenesis, single channel analysis, and identification of native molecules in TRP channel complexes (see [38]). In contrast, simpler experiments could assess whether citral blocks the open pore of TRPV4 and TRPA1 channels.

One of the important disagreements in the literature of TRP channels concerns the presence and distribution of TRPV1–3 channels in dorsal root ganglion neurons. Usually of low to moderate abundance, ion channel distributions are more reliably determined by the exquisitely sensitive and specific patch clamp technique rather than by immunohistochemistry. Challenges in the TRP field have been the lack of specific toxins, blockers, and activators, low amplitudes of TRP currents, similarity of TRP current-voltage relations, lack of cation selectivity, and a dearth of reliable, specific antibodies. In this study, we made use of the citral's partial agonist effects and tools developed in other studies. We showed in heterologous expression that citral's prolonged inhibitory effects could eliminate activated TRPV1 current. The remaining TRPV2 or TRPV3 channels could then be separated. TRPV2 currents were not potentiated by repeated 2-aminoethyldiphenyl borate application, while camphor activated TRPV3 and the channels were sensitized by repeated 2-aminoethyldiphenyl borate applications. Although 2-aminoethyldiphenyl borate is a notoriously nonspecific blocker of many channels and transporters, it relatively specifically activates TRPV1–3 [39], [40] and TRPA1 [41] in the 0.1 mM range of concentrations. TRPA1 is present in a low percentage of dorsal root ganglion neurons, however, and its activity should have been apparent upon citral washout.

Using citral and 2-aminoethyldiphenyl borate as pharmacological tools, we found that the majority of cells from juvenile rat dorsal root ganglion neurons expressed functional TRPV1, TRPV2, and TRPV3 channels. In addition, antibody labeling was consistent with widespread expression of TRPV2 in rat postnatal day 12–20 acutely isolated dorsal root ganglion neurons. In contrast, previous immunohistochemical study of adult rats indicated that TRPV2 was in <20% of dorsal root ganglion neurons [28]–[30]) and TRPV1 colocalized with TRPV2 in <30% of the TRPV1-positive population [29], [42], [43]. TRPV1–3 levels may be developmentally regulated, or identification and separation of functional TRPV currents in adult dorsal root ganglion neurons may modify previous estimates.

Citral is a well-known odorant, and is commonly assumed to act primarily via G protein coupled receptors in olfactory epithelia. Our results raise the question of whether it also affects odor sensation via the TRP channels studied here. Indeed, a number of molecules that activate TRP channels are used as odorants in olfactory testing and neuronal mapping studies [6], [44], [45]. Several TRP channel proteins are expressed in sensory fibers of the trigeminal ganglia [13], [17], [24], [26], [28], [36], [46] and nasal epithelium [16]. With increased understanding of TRP channel distribution, there is renewed interest in signaling between epithelia and the dorsal root ganglion neurons underlying somatosensation [47]. Additionally, TRP channels are commonly potentiated by Gq/11 mediated G protein coupled receptors. These points should be taken into consideration in mapping odorant signaling from nose to brain, since GPCR and TRP channel subtype distributions are likely to be distinct.

Citral's medicinal effects from ancient to modern times may be explained by some of the actions we report here, but it is difficult to parse the anecdotal and uncontrolled evidence over the millennia of its use. More thoroughly studied, topical capsaicin can be useful for some types of pain [48]–[51]. Interestingly, co-application of anandamide and capsaicin evoked TRPV1-like currents in mouse trigeminal neurons that were significantly smaller than currents evoked by capsaicin alone [52]. Thus, partial TRPV1 agonists might be useful in anti-inflammatory and analgesic compounds to attenuate capsaicin's fiery sting. Similar to capsaicin, citral's low potency limits its use to topical application. However, citral's broad spectrum and prolonged sensory inhibition may prove more useful than capsaicin for allodynia, itch, or other types of pain involving superficial sensory nerves and skin.

## Materials and Methods

### Cell culture and transfection

Transformed human embryonic kidney-293 (HEK-293T) cells were grown in Dulbecco's modified Eagle's medium (DMEM)+F-12 supplemented with 10% fetal calf serum, and 1% penicillin/streptomycin in 5% CO_2_ at 37°C. Cells grown to 90% confluence in 35 mm Petri dishes were transiently transfected with plasmid DNA encoding a TRP channel construct (1 µg). Rat TRPV1 (rTRPV1) was cloned into an enhanced green fluorescent protein-containing vector (pTracer-CMV2; Invitrogen, Carlsbad, CA). Chicken TRPV1 (cTRPV1), the chicken rat TRPV1 chimera (crcTRPV1), rat TRPV2 (rTRPV2), mouse TRPV3 (mTRPV3), mouse TRPV4 (mTRPV4), rat TRPM8 (rTRPM8), and rat TRPA1 (rTRPA1), were cloned into pcDNA3 and co-transfected with enhanced green fluorescent protein (0.1 µg) to follow expression. The cTRPV1 and crcTRPV1 clones were kindly provided by Dr. David Julius [31]. Recordings were carried out 24–36 hours after transfection. Cells were split via trypsin EDTA, plated on glass coverslips at 5–10% confluence, and used 2–3 hours later.

### Preparation of dorsal root ganglion neurons

Dorsal root ganglion neurons from all spinal levels were removed from postnatal day 12 to 20 Sprague Dawley rats and the nerve roots trimmed. Calcium- and magnesium-free (CMF) Hank's solution contained (in mM): 136.9 sodium chloride, 5.4 potassium chloride, 0.34 dibasic sodium orthophosphate, 0.44 monopotassium phosphate, 5.6 glucose, 5 4-(2-hydroxyethyl)-1-piperazineethanesulfonic acid, 0.005% phenol red, pH 7.4. CMF Hank's was used throughout the isolation and enzymatic treatments. The isolated ganglia were treated for 20 minutes at 37°C with 20 U/ml of papain (Worthington Biochemical, Lakewood, NJ) and 5 mM cysteine. The ganglia were then transferred to CMF Hank's with 3 mg/ml collagenase (type I; Worthington) and 4 mg/ml dispase (neutral type II; Roche Applied Science, Indianapolis, IN) for 20 minutes at 37°C. Individual neurons were dispersed by trituration through a fire-polished glass Pasteur pipette in 3 ml of media containing DMEM, 5% horse serum, 10% fetal bovine serum, 1% penicillin/streptomycin, and 100 ng/ml Nerve Growth Factor (7S subunit; Invitrogen). The neurons were preplated in tissue culture-treated Petri dishes for 1 hour when used for electrophysiology (3 hours for calcium imaging), and then plated on poly-L-lysine (1 mg/ml)-coated glass coverslips. Dorsal root ganglion neurons were allowed to settle for 1–2 hours at 37°C before patching or imaging over the next 6 hours.

### Calcium imaging

Dorsal root ganglion neurons were loaded with 5 µM fura-2 AM and 0.02% pluronic acid in media at 37°C for 60 minutes. Cells were then washed with modified Ringer's solution with low [Cl^−^] and 2 mM calcium (see below). The MetaFluor imaging system (Universal Imaging Corporation) was used to record fluorescence at 340 and 380 nm excitation wavelengths. Background fluorescence, measured as the ratio 340/380 in a “cell-free” zone during the experiment, was subtracted from the data. The ratio of the wavelengths (F340/F380) reflects changes in the internal calcium concentration after perfusion with the indicated compounds.

### Immunocytochemistry

Antisera to TRPV2 were raised against a carboxy-terminal peptide (KNSASEEDHLPLQVLQSH) and affinity purified on a Sulfolink (Pierce) column. Cells were fixed in 4% paraformaldehyde sucrose solution (15 minutes), washed, permeabilized with a 2% bovine serum albumin, fetal bovine serum, and fish gelatin solution with 0.2% triton-X (15 minutes), and incubated in blocking solution (30 minutes). Anti-TRPV2 antibody was added at 0.3 µg/ml (1 hour); for control experiments the antibody was preincubated with 10× blocking peptide. Coverslips were washed and secondary antibodies were applied (1 hour). Mouse anti-rabbit Alexa-488 (1∶2000; Invitrogen) secondary was used for dorsal root ganglion neurons; goat anti-rabbit Alexa-647 was used for experiments with expressed enhanced green fluorescent protein-tagged channels. Controls were labeled with secondary antibody alone. Coverslips were again washed, mounted on slides (Fluoromount G; EMS), and imaged with a confocal microscope at identical settings for the test and control groups.

### Biochemistry

10 centimeter dishes of 293T cells transfected with tagged TRPV2 (enhanced green fluorescent protein- or hemagglutinin A-; 3 µg/µl of each) were treated with Ringer's (control), 2-aminoethyldiphenyl borate (700 µM), or 2-aminoethyldiphenyl borate +citral (2 mM) for 10 minutes, rinsed with ice-cold phosphate buffer solution, harvested and lysed (500 µl 1% triton-X). Immobilized anti-hemagglutinin A beads (20 µl) were added to the supernatant overnight. The western blot of the eluted proteins was exposed to rabbit anti-TRPV2 antibody (0.1 µg/ml) and anti-rabbit horseradish peroxidase secondary. Chemilumenescence was assessed using a Fuji imager.

### Electrophysiology

Electrophysiological experiments were performed using the whole-cell patch-clamp technique at room temperature. Modified Ringer's solution with low chloride and nominal calcium contained (in mM): 130 sodium hydroxide, 5 potassium chloride, 1 magnesium chloride, 10 sodium 4-(2-hydroxyethyl)-1-piperazineethanesulfonic acid, 10 2-(N-morpholino)ethanesulfonic acid, and 20 glucose (pH 7.4; 310 milli-osmolar). Nominal zero calcium (<10 µM) significantly reduced desensitization of TRP channels. 2 mM calcium chloride was included in the external solution where noted. To activate TRPV1, the modified Ringer's solution was made at pH 5 (low pH solution) and the chloride concentration was kept to a minimum to prevent contamination of the TRPV1 currents with acid-activated chloride currents. For recordings from heterologously expressing cells, the internal pipette solution contained (in mM): 125 cesium-Methanesulfonate, 10 cesium (1.2-bis(o-aminophenoxy)ethane-N,N,N',N'-tetraacetic acid, 8 sodium chloride, 4 magnesium adenosine triphosphate, and 10 HEPES (pH 7.2; 290 mOSM; nominal free calcium concentrations). For TRPA1 and dorsal root ganglion recordings, the internal pipette solution contained (in mM): 110 cesium-Methanesulfonate, 0.5 cesium_4_BAPTA, 8 sodium chloride, 0.2 calcium chloride, 4 magnesium-ATP, 4 sodium-GTP, and 10 4-(2-hydroxyethyl)-1-piperazineethanesulfonic acid (pH 7.2; 290 mOSM; 100 nM free calcium internal). MaxChelator was used to calculate the free calcium concentration (http://www.stanford.edu/cpatton/maxc.html).

Recordings were obtained using an Axopatch 200B amplifier, Digidata 1322A analog-to-digital converter, and pClamp 8.01 software (Molecular Devices, Union City, CA). Data were low-pass filtered at 2 kHz and digitized at 5 kHz. Borosilicate glass pipettes (World Precision Instruments, Sarasota, FL) were typically 3 MΩ pipette resistance after fire polishing. Cell capacitance was measured for each cell, and access resistance compensated to ∼80%. Recordings were begun ∼5 minutes after initial rupture to ensure initial steady state conditions. Current-voltage (I-V) relations were obtained using a ramp protocol. Voltage ramps (400 ms) from −100 to +40 or +100 mV were applied every 1–2 seconds and the holding potential was either −70 mV or 0 mV (see Figure Legends). A 40 ms step was added at the beginning and end of the ramp (i.e. at −100 mV, +40 mV or +100 mV). Liquid junction potentials were not corrected.

### Data analysis

Citral activation dose response curves were generated by normalizing peak currents evoked by a given [citral] to the peak current evoked by the highest [citral] shown. Citral inhibition dose response curves were generated by averaging the percentage block of agonist-evoked TRP current at each [citral]. The dose response curves were fit with the Hill equation, where 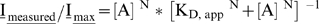 and [A] is the concentration of agonist or blocker, N is the Hill coefficient, and K_D_ is the [A] for I_measured_/I_max_ = 0.5. The term apparent dissociation constant is used in place of the half concentration of excitation and inhibition. Overexpression of rTRPA1 resulted in constitutive currents, which the citral rTRPA1 response was normalized to. Averaged data are presented as mean±standard error (S.E.) throughout. Statistical analysis included paired and unpaired Student's t-test comparisons of the data; p values are reported in the text.

### Reagents

Citral, geraniol, capsaicin, menthol, allyl isothiocyanate, 4α-phorbol-12, 13-didecanoate, and 2-aminoethoxydiphenyl borate were obtained from Sigma (St. Louis, MO). Nerol was obtained from Spectrum Chemicals (Gardena, CA). Geranial and neral were obtained through o-iodoxybenzoic acid (IBX) oxidation of the alcohols geraniol and nerol [53]. Geraniol (500 mg, 3.24 mM) was dissolved in ethyl acetate (15 mL), and IBX (1.82 g, 6.48 mM) was added. The resulting suspension was refluxed for 1.5 hours, after which the reaction was cooled to 0°C and diethyl ether (15 mL) was added. The suspension was filtered, the filter cake was washed with ethyl acetate (2×10 mL), and the combined filtrates were concentrated by rotary evaporation. Flash chromatography (1∶1 diethyl ether/hexanes) yielded geranial as a clear oil (436 mg, 88%). ^1^H NMR (CDCl_3_, 300 MHz) δ 9.99 (d, 1H, J = 8.1 Hz), 5.89 (d, 1H, J = 8.1 Hz), 5.06 (m, 1H), 2.25–2.16 (m, 7H), 1.69 (s, 3H), 1.61 (s, 3H) [54]. Nerol (500 mg, 3.24 mM) was oxidized in the same manner as for geranial, but the suspension was refluxed for 18 hours. Flash chromatography (1∶1 diethyl ether/hexanes) yielded neral as a light yellow oil (487 mg, 99%). ^1^H NMR (CDCl_3_, 300 MHz) δ 9.89 (d, 1H, J = 8.1 Hz), 5.88 (d, 1H, J = 8.1 Hz), 5.10 (1H, m), 2.58 (t, 2H, J = 7.5 Hz), 2.23 (q, 2H, J = 7.5 Hz), 1.99 (s, 3H), 1.68 (s, 3H), 1.59 (s, 3H) [55]. Stock solutions were made in dimethyl sulfoxide. The final concentration of dimethyl sulfoxide in working solutions did not exceed 0.001%, a concentration that had no effect in control experiments.

## Supporting Information

Figure S1 (5.82 MB TIF)

Figure S2 (4.80 MB TIF)

Figure S3 (7.34 MB DOC)

Figure S4 (7.08 MB TIF)

Figure S5 (10.30 MB TIF)
