# Correction: Citral Sensing by Transient Receptor Potential Channels in Dorsal Root Ganglion Neurons

**DOI:** 10.1371/annotation/6ba8e9d9-0035-405e-a7c7-45ee22b2e381

**Published:** 2008-05-13

**Authors:** Stephanie C. Stotz, Joris Vriens, Derek Martyn, Jon Clardy, David E. Clapham

There was an error in the published title of this manuscript. The correct title is: "Citral Sensing by Transient Receptor Potential Channels in Dorsal Root Ganglion Neurons."

