# Supplementary figures and images for: Citral Sensing by TRANSient Receptor Potential Channels in Dorsal Root Ganglion Neurons
Source: PLoS One. 2008 May 7;3(5):e2082. doi: 10.1371/journal.pone.0002082 (PMC2346451; doi:10.1371/journal.pone.0002082)

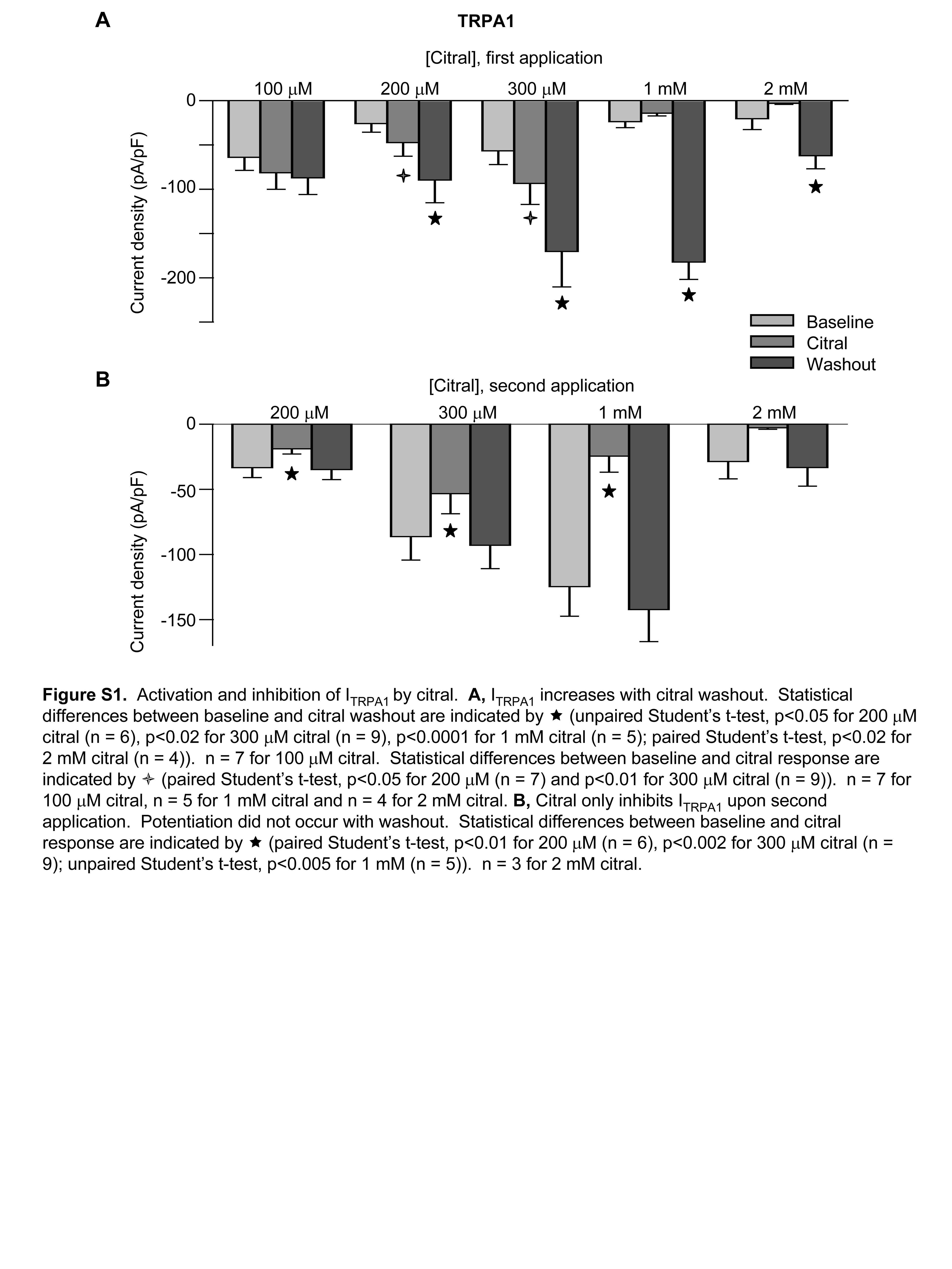

Supplement: Figure S1 — (5.82 MB TIF) [file pone.0002082.s001.tif]

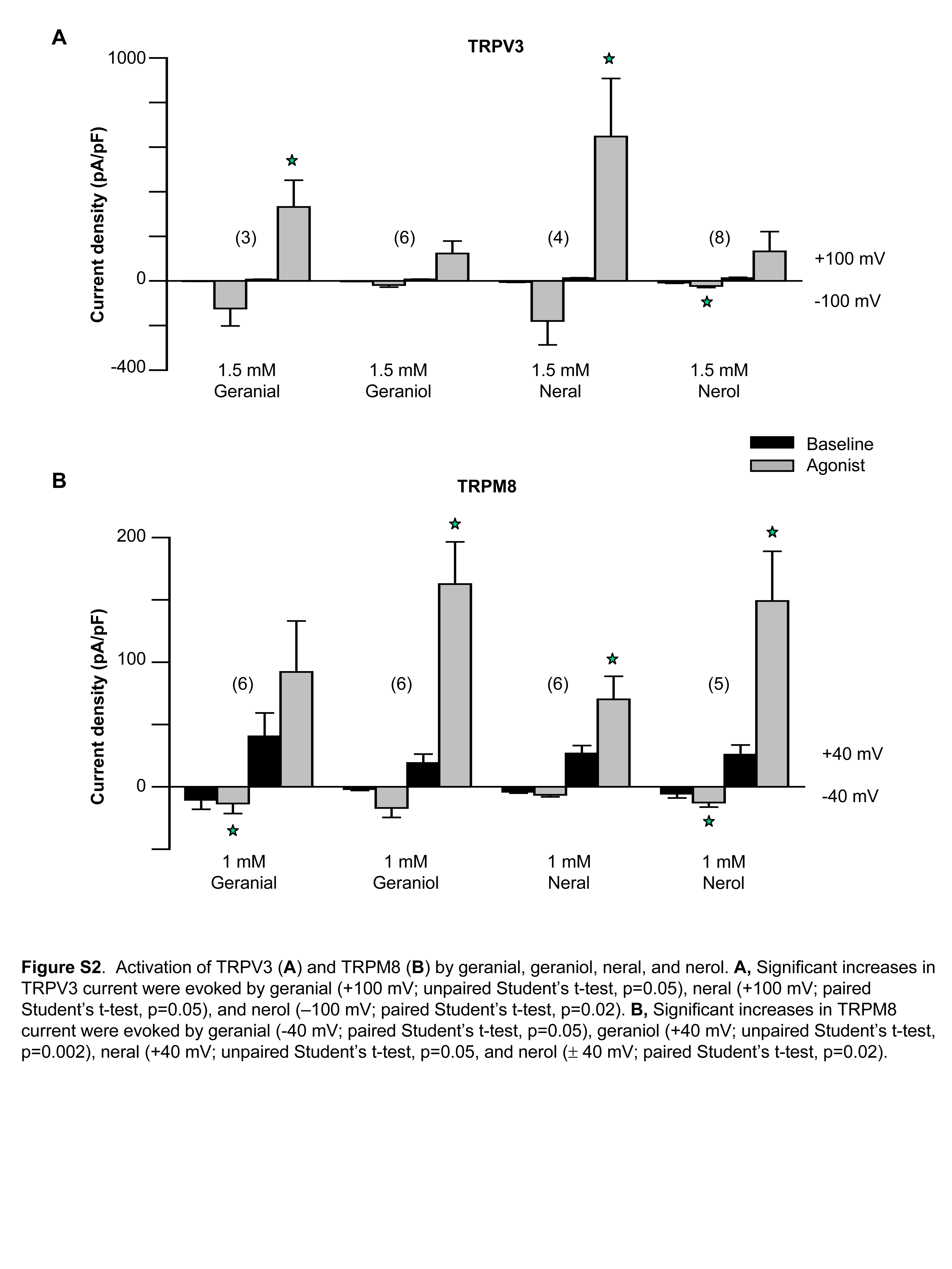

Supplement: Figure S2 — (4.80 MB TIF) [file pone.0002082.s002.tif]

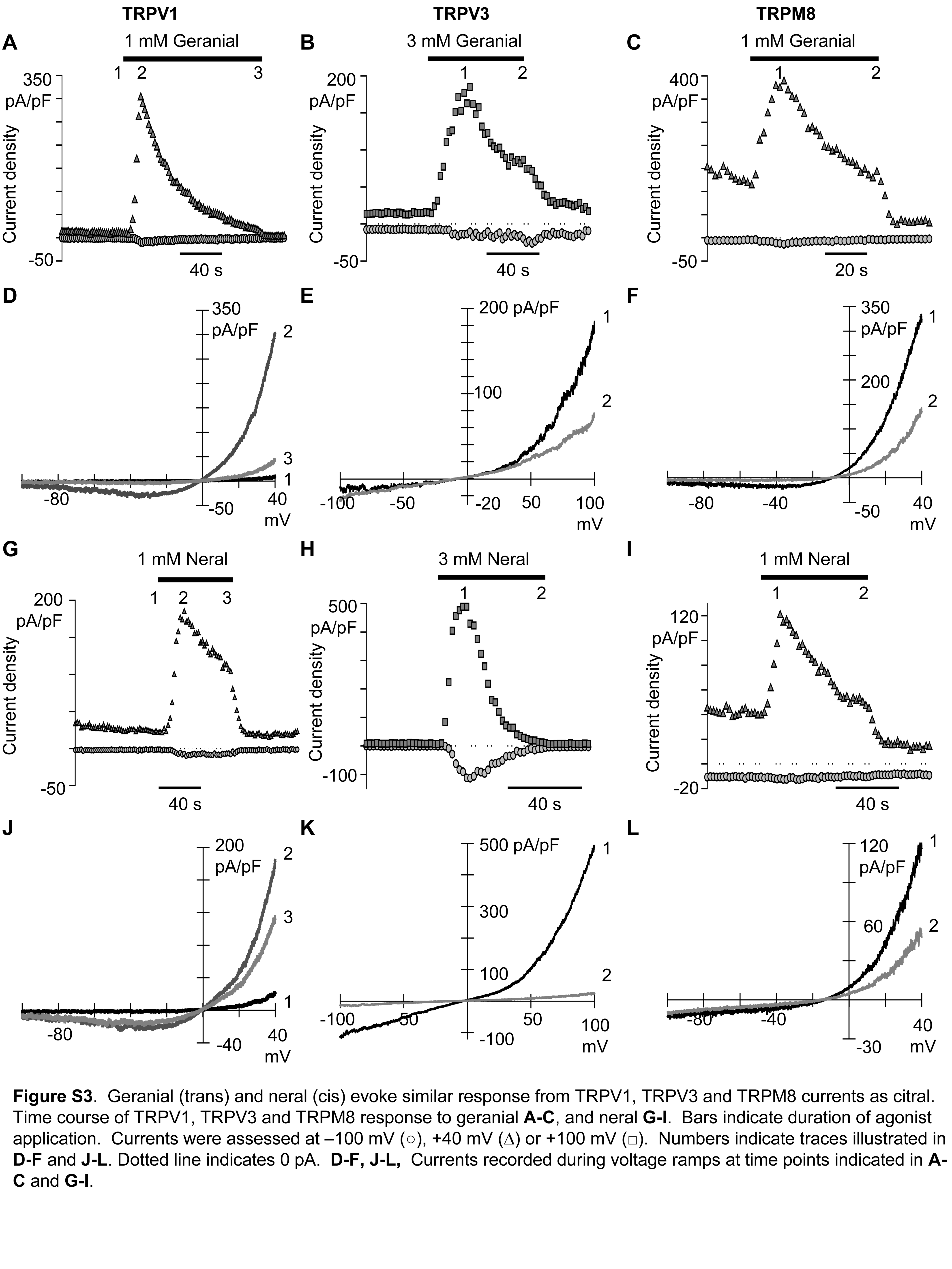

Supplement: Figure S3 — (7.34 MB DOC) [file pone.0002082.s003.tif]

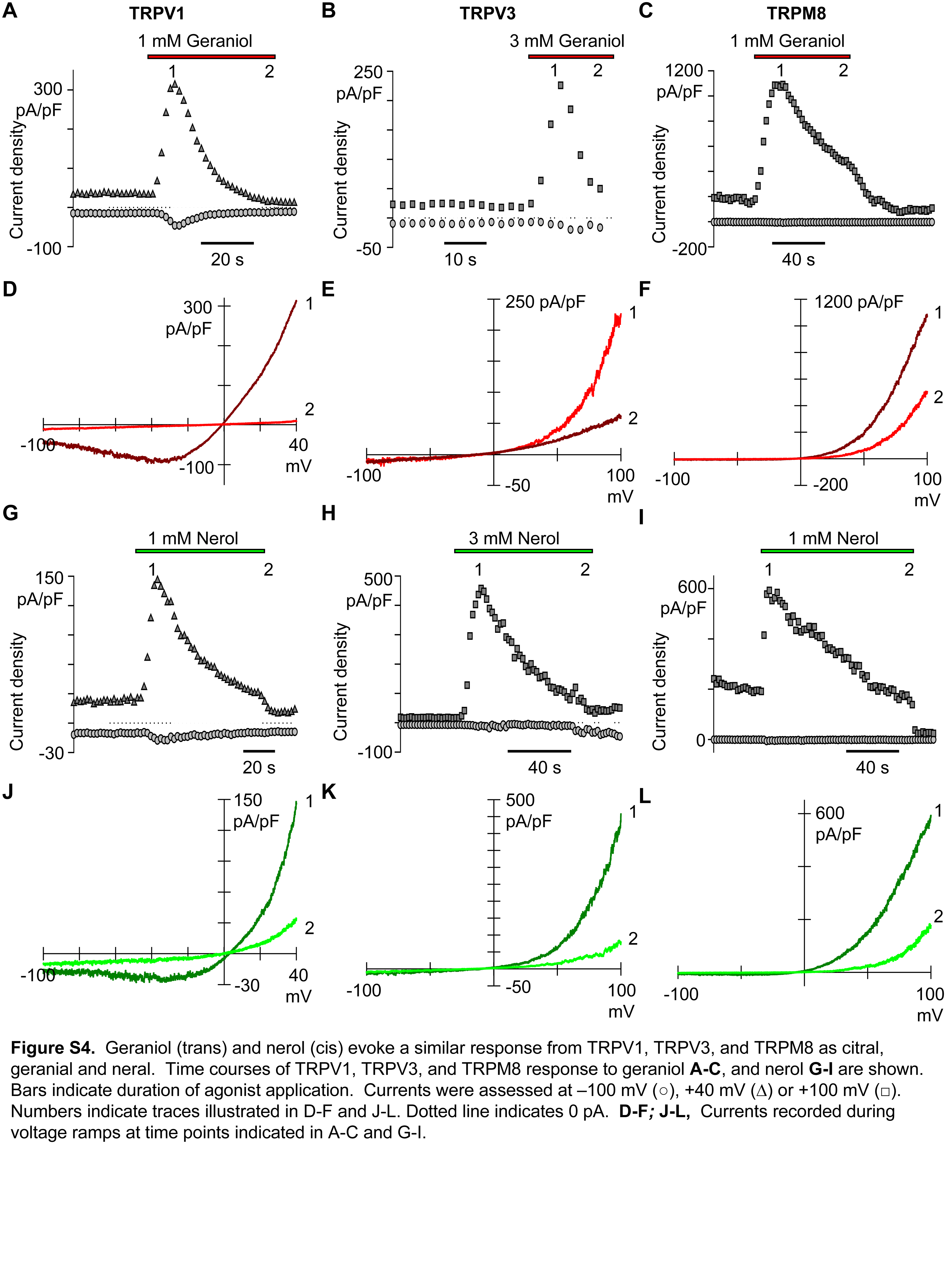

Supplement: Figure S4 — (7.08 MB TIF) [file pone.0002082.s004.tif]

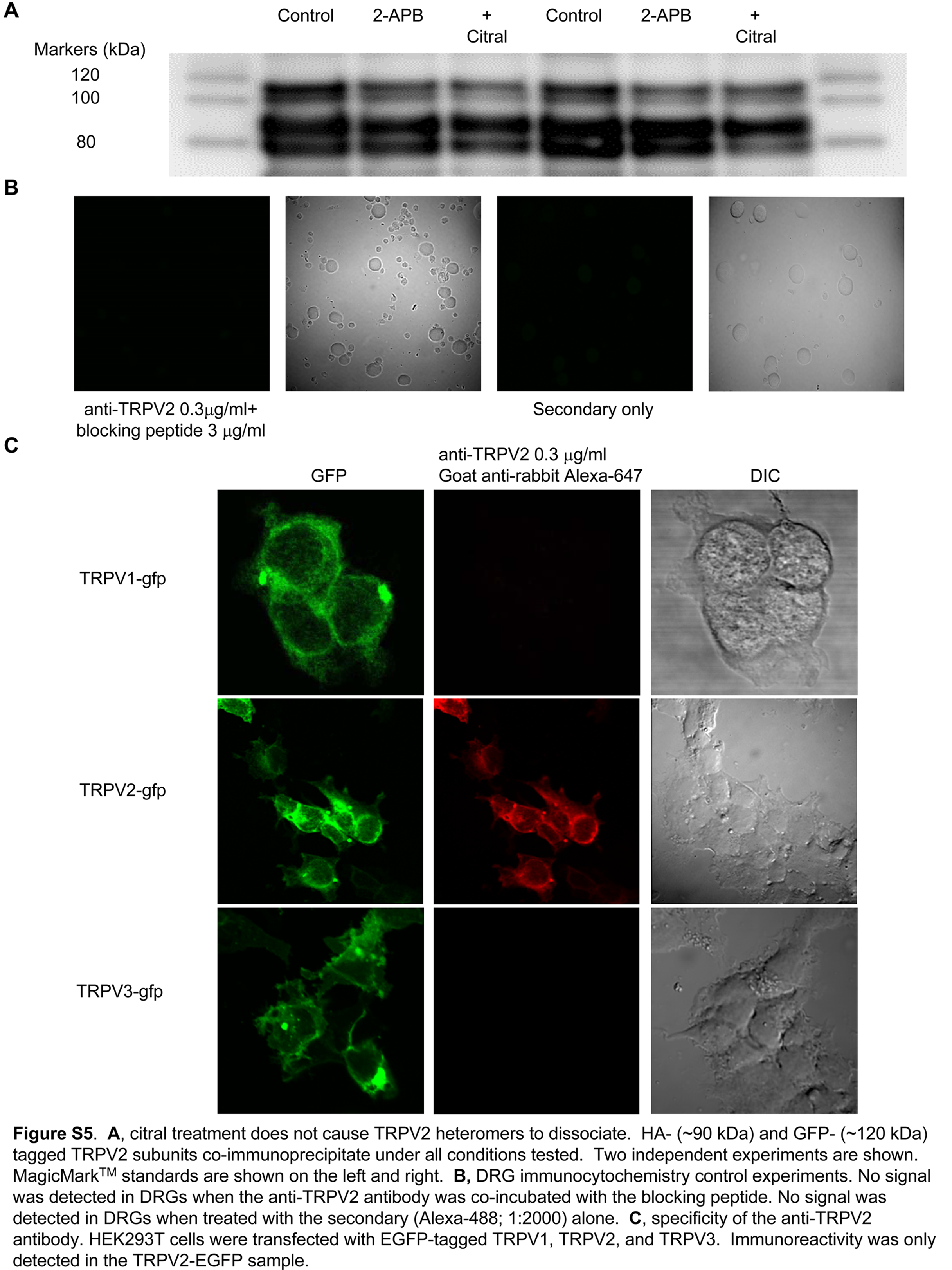

Supplement: Figure S5 — (10.30 MB TIF) [file pone.0002082.s005.tif]
